# Intravenous calcium as a pressor in a swine model of hypoxic pseudo-pulseless electrical mechanical activity—a preliminary report

**DOI:** 10.1186/s40635-020-00340-0

**Published:** 2020-09-04

**Authors:** Alexander L. Lindqwister, Joshua W. Lampe, Jeffrey R. Gould, Christopher L. Kaufman, Karen L. Moodie, Norman A. Paradis

**Affiliations:** 1 Geisel School of Medicine, Dartmouth College, Hanover, NH 03775 USA; 2 ZOLL Medical Corp, Chelmsford, MA 01824 USA; 3 Geisel School of Medicine, Dartmouth Hitchcock Medical Center, 1 Medical Center Dr #4B, Lebanon, NH 03756 USA

**Keywords:** Pressor, Calcium, PEA, Pulseless electrical activity

## Abstract

**Background:**

Pseudo-pulseless electrical activity (pseudo-PEA) is a lifeless form of profound cardiac shock characterized by measurable cardiac mechanical activity without clinically detectable pulses. Pseudo-PEA may constitute up to 40% of reported cases of cardiac arrest. Resuscitation from pseudo-PEA is often associated with hypotension refractory to catecholamine pressors. We hypothesized that this post-resuscitation state may be associated with hypocalcemic hypotension responsive to intravenous calcium.

**Methods:**

Using pre-existing data from our hypoxic swine pseudo-PEA model, we measured blood pressure, hemodynamics, and electrolytes. Physiological data were analyzed on a heartbeat by heartbeat basis. The midpoint of the calcium response was defined using change of curvature feature detection. Hemodynamic parameters were shifted such that the value at the midpoint was equal to zero.

**Results:**

In 9 animals with refractory hypotension, we administered 37 boluses of intravenous calcium in the dosage range of 5-20 mg. Comparisons were made between the average values in the time period 40-37 s before the midpoint and 35-40 s after the midpoint. Of the 37 administered boluses, 34 manifested a change in the blood pressure, with mean aortic pressure, systolic and diastolic pressures all increasing post bolus administration.

**Conclusions:**

Administration of intravenous calcium may be associated with a pressor-like response in refractory hypotension after resuscitation from pseudo-PEA. Relative ionized hypocalcemia may cause hypotension after resuscitation from pseudo-PEA. Therapy with intravenous calcium should be further investigated in this setting.

## Introduction

The post-resuscitation state is often characterized by hypotension that is refractory to pressors [1]. While it is likely that the myocardial and vasomotor processes are multifactorial, it has been proposed that disordered calcium homeostasis is a central factor [2, 3], as ionized calcium plays an important role in both inotropy and vasomotor tone [4].

Nieman et al. previously reported that ionized hypocalcemia occurs post return of spontaneous circulation (ROSC) in their swine model of ventricular fibrillation [2, 3], and that administration of intravenous (IV) calcium gluconate is associated with a transient improvement in hemodynamics.

Historically, clinical cardiac arrest has been classified into two broad categories that include ventricular fibrillation and pulseless electrical activity (PEA). The latter category was first called electromechanical dissociation (EMD), but underwent a name change when studies revealed that in a substantial fraction of patients, pulse pressures and ventricular mechanical function were detectible [5, 6]. The detection of pulses with advanced technology renders PEA an imprecise descriptor, and we now refer to the cardiovascular state of subjective clinical cardiac arrest with detectable hemodynamics as “pseudo-PEA.” Specifically, pseudo-PEA is a global hypotensive ischemic state with retained coordinated myocardial contractile activity and an organized ECG.

Similar to ventricular fibrillation, pseudo-PEA causes a profound global ischemia and is often associated with post-resuscitation hypotension [7]. Extending Nieman’s report of IV calcium responsive hypotension after resuscitation from fibrillation cardiac arrest, we now provide a preliminary report of a calcium pressor response in hypotension after resuscitation from pseudo-PEA.

## Methods

The manuscript contains a post hoc analysis of data originally obtained in related studies using our established pseudo-PEA model [8, 9]. These studies were conducted in accordance with the guidelines of the National Research Council of the National Academies and with the approval of the Dartmouth College Institutional Animal Care and Use Committee. The model utilized was a variation on previously described porcine asphyxial P-EMD preparation [5, 6, 10].

### Surgical prep

Three cohorts (*N* = 38) of domestic farm raised Yorkshire swine weighing approximately 30 kg were fasted overnight with free access to water and then sedated with an intramuscular injection of ketamine (30 mg/kg). After endotracheal intubation, anesthesia was initiated and maintained with isoflurane (0.5-4%) and oxygen (1-3 L/min). During preparation, ventilation was provided by a volume-controlled ventilator (GE Datex-Ohmeda Modulus SE, Madison, WI) with 100% O_2_ (tidal volume of 15-20 cc/kg and ventilation rate of 8-15 breaths per minute) during initiation, reducing to 30% shortly thereafter. Ventilation rate and tidal volume were initially adjusted to maintain normocapnia (the end-expiratory partial pressure of CO_2_ between 35-45 mmHg) as measured continuously by a capnometer (CO2SMO, Novametrix, Wallingford, CT) placed in the airway. Arterial blood gases (I-Stat, Abbott Point of Care, Princeton, NJ) were analyzed to confirm adequate baseline ventilation. Throughout the experiment, the animals were monitored using ECG, end-tidal CO_2_, and arterial blood pressure. In addition, depth of anesthesia was continuously assessed.

The animals were secured in a supine position and were given normal saline at a rate of 10 ml/kg per hour through a vein to maintain a central venous pressure of ~ 5 mmHg. Through either ultrasound-guided percutaneous cannulations or surgical cut-down, micromanometer catheters with a lumen were placed into (1) the right atrium via the femoral vein, and (2) the descending aorta through the femoral artery for pressure measurements. Cohort 1 utilized Tru-Wave Pressure Transducers (Edwards Lifesciences, Irvine, CA), and cohorts 2 and 3 utilized SPR-350 (Millar Instruments, Houston, TX). All catheters were positioned under fluoroscopic guidance, and unfractionated heparin (100 units/kg) was given to prevent catheter clotting. Flow probes were placed around the carotid artery (3 PS probe, Transonic, Ithaca, NY) and jugular vein (2.5 PS probe, Transonic, Ithaca, NY) via a cut-down procedure.

After instrumentation, baseline measurements were obtained for all variables including blood gas analyses. Analog outputs of the physiological parameters were digitized and stored in data files on a personal computer for further analysis using a 16-channel computerized data-acquisition system at a sampling rate of 1000 Hz (Powerlab 16SP, ADInstruments, Castle Hill, Australia). Raw data channels included ECG, aortic pressure, right atrial pressure, intra-cranial pressure, capnography, carotid blood flow, and jugular blood flow.

### Pseudo-PEA induction and chest compression

Animals were converted to continuous intravenous anesthesia using ketamine (50 mcg/kg/min) and fentanyl (0.45 mcg/kg/min), isoflurane was gradually discontinued. Depth of anesthesia was ensured via monitoring blood pressure, heart rate, and jaw tone. The IV anesthesia protocol was maintained 15 min to allow isoflurane washout and to establish a stable level of continuous IV anesthesia prior to initiation of the hypoxia protocol.

Arterial blood gases were measured at baseline and after each episode of pseudo-PEA (i-STAT, Abbott Point of Care, Abbott Park, IL). Measurements included: pH, pCO2, pO2, base excess, HCO3, TCO2, O2 percent saturation, Na (mmol/L), K (mmol/L), ionized calcium (iCa), glucose, hematocrit, and hemoglobin.

Once adequate anesthesia has been confirmed, the animals were paralyzed using vecuronium (1.0 mg/kg) to minimize gasping [11]. Baseline data were measured before any injury occurred. Round 1 data were measured after resuscitation from the first pseudo-PEA injury. Data were also measured after resuscitation from subsequent pseudo-PEA injuries. The number of injuries per animal is variable, but the maximum number of hypoxic episodes is 5. Animals were ventilated with a progressively hypoxic gas mixture of O_2_/N_2_. Gas concentrations were measured using an oxygen concentration analyzer (Oxygen Analyzer S-3A/II, Applied Electrochemistry, VMETEK) and the concentration of O2 was decreased until pseudo-PEA was achieved. Onset of pseudo-PEA was defined as sustained aortic systolic pressure ≤ 50 mmHg recorded by the aortic catheter in the presence of an organized cardiac rhythm.

Animals were treated with mechanical chest compressions delivered at a depth of 5 cm and at a variable rate. Compressions were delivered for a total of 6 min. After 6 min of chest compressions, the FiO2 was set to 100%, and chest compressions were continued until ROSC was achieved. ROSC was defined as a systolic pressure > 60 mmHg without chest compressions. If ROSC was detected at any point during the chest compressions with ongoing hypoxia, compressions were terminated and FiO2 was set to 100%.

### Post resuscitation treatment

Arterial blood gases were measured 10 min after ROSC in all animals and for all episodes of pseudo-PEA. Three related experiments that utilized the same method of hypoxic injury and resuscitation were performed on three separate cohorts of swine (Tables 1 and 2). Animals in cohorts 1 and 2 did not receive ionized calcium. The observation that ionic calcium concentration decreases following pseudo-PEA led to the addition of Ca gluconate to the treatment regimen post-resuscitation for the 14 animals in cohort 3. Animals in cohort 3 were administered ionized calcium in an ad hoc fashion during the recovery phase when MAP was not sufficient or stable. A bolus of Ca gluconate would be delivered intravenously if the aortic blood pressure was decreasing after resuscitation. This meant that calcium might be given to treat blood pressures before the blood chemistries were drawn, resulting in some missing data.

**Table 1:** Results of cohorts 1 and 2 of animal experiments

	Animal	Baseline		Round 1		Round 2		Round 3		Round 4		Round 5	
		pH	iCa	pH	iCa	pH	iCa	pH	iCa	pH	iCa	pH	iCa
**Cohort 1**	1	7.46	1.29	7.29	1.21	7.32	1.12	7.31	1.1	7.28	1.17	7.29	1.20
	2	7.50	1.26	7.27	1.21	7.23	1.24	7.37	1.23	7.34	1.28	7.35	1.26
	3	7.47	1.28	7.32	1.24	7.32	1.25	7.29	1.24	7.32	1.27	7.4	1.25
	4	7.49	1.29	7.25	1.20	7.20	1.25	7.24	1.18	7.29	1.15	7.24	1.17
	5	7.50	1.35	7.53	1.33	7.48	1.26	7.51	1.26	7.49	1.25	7.31	1.25
	6	7.49	1.36	7.46	1.25	7.46	1.22	7.39	1.25	7.36	1.28	7.42	1.72
	7	7.48	1.39	7.51	1.34	7.48	1.33	7.51	1.30	7.50	1.28	7.50	1.28
	8	7.51	1.41	7.33	1.29								
	9	7.50	1.40	7.40	1.07	7.44	1.27	7.45	1.28	7.38	1.28		
	10	7.47	1.36	7.09	1.28								
	11	7.48	1.33	7.24	1.32	7.27	1.25						
	12	7.50	1.17	7.33	1.27	7.33	1.27						
	13	7.48	1.29	7.33	1.25	7.38	1.22						
	14	7.48	1.30	7.33	1.32								
**Cohort 2**	1	7.24	1.19										
	2	7.23	1.20										
	3	7.49	1.23	7.32	1.14								
	4	7.48	1.29	7.48	1.29								
	5	7.51	1.13										
	6	7.43	1.17										
	7	7.45	1.33	7.25	1.21	7.33	1.11	7.31	1.16				
	8	7.42	1.07	7.23	1.07	7.28	0.92	7.28	0.88				
	9	7.48	1.34	7.34	1.17	7.33	1.11	7.32	1.11	7.34	1.08		
	10	7.45	1.23	7.32	1.11	7.20	0.92	7.20	1.06

**Table 2:** Results of cohort 3 of animal experiments

	Animal	Baseline		Round 1				Round 2				Round 3				Round 4			
		pH	iCa	pH	iCa	Bolus Ca	Epi	pH	iCa	Bolus Ca	Epi	pH	iCa	Bolus Ca	Epi	pH	iCa	Bolus Ca	Epi
**Cohort 3**	1	Missing	Missing	Missing	Missing	0	1	Missing	Missing	0	0								
	2	7.49	1.08	7.16	1.21	0	1	7.50	0.94	0	0	7.37	0.92	0	0	7.22	1.05	0	0
	3	7.45	1.38	7.47	1.38	0	6	7.04	1.37										
	4	7.62	1.25	7.42	1.39	0	0	7.16	1.29	1	0	7.39	1.12	0	1	7.30	1.17	0	1
	5	7.44	1.34	7.01	1.26	1	0	7.28	1.42	1	0	7.40	1.25	1	0	7.21	1.22	0	0
	6	7.47	1.44	7.20	1.25	0	0	7.32	1.29	1	0	7.24	1.49	2	0	Missing	Missing	0	0
	7	7.45	1.38	7.03	0	0	5												
	8	7.47	1.40	7.44	1.30	3	0	7.40	1.24	3	0	7.34	1.36	2	0	Missing	Missing	1	2
	9	7.46	1.25	7.47	1.33	1	1	7.28	1.22	5	0	7.40	1.30	5	0	7.30	1.72	0	1
	10	7.49	1.39	7.01	0.87	2	0	7.30	1.48	1	1	7.29	1.40	3	0	7.39	1.37	0	0
	11	7.44	1.39	7.29	1.32	0	0	7.29	1.27	3	3	Missing	Missing	1	3	Missing	Missing	0	0
	12	7.44	1.33	7.29	1.23	1	0	Missing	Missing	0	0								
	13	7.43	1.47	7.26	1.23	0	0	7.14	1.27	3	2	Missing	Missing	0	0				
	14	7.45	1.36	7.35	1.24	0	0	7.48	1.18	0	0	7.47	1.11	0	0	Missing	Missing	0	0

Three cohorts of swine underwent related experiments using the same method of hypoxic injury. Cohorts 1 (*N* = 48 total rounds) and cohort 2 (*N* = 15 total rounds) did not receive ionized calcium treatments. Cohort 3 (*N* = 37 Ca++ boluses) received ad hoc ionized calcium in 9 of the 14 experiments. Cohorts 2 and 3 underwent a maximum of 4 experimental rounds, whereas cohort 1 underwent a maximum of 5. Blank entries indicate the animal did not survive to that round of experiment. Some blood gas data was missing, which is labeled accordingly

In addition, epinephrine and sodium bicarbonate were also delivered to support perfusion pressures and correct post-resuscitation blood chemistry as needed. As a result, different animals received different numbers and dosages of calcium boluses. Individual animals received as many as five periods of hypoxia-induced pseudo-PEA followed by resuscitation. Time of delivery of epinephrine, bicarbonate, and calcium were annotated in the physiological data file from each experiment.

### Data selection

Of the 14 animals in cohort 3, 9 received calcium injections, for a total of 37 boluses of calcium. For each recorded calcium bolus, a data subset was exported that included time intervals before and after the bolus. Because the calcium was delivered in an un-protocolized manner, the amount of time before (or after) the bolus varied for each calcium injection. In the event that a calcium injection was preceded or followed by a different injection of calcium or injections of bicarbonate or epinephrine, the data subset started (or ended) half-way between the two injections. This was done so as to isolate the effect of the injection of calcium from the effects of other injections. One critical limitation of this method is that there was significant variation in the amount of time before and after a calcium injection. The minimum amounts of time before and after each calcium injection was 40 s and our analysis focused on these intervals.

### Data analysis

Individual heartbeats were identified using a python script (Anaconda 1.9.6, Spyder version 3.1.2, Pandas version 0.22.0, Numpy version 1.14.0). Once the location of each heartbeat was identified, the mean arterial pressure (MAP), the systolic aortic pressure (AoS), and the diastolic aortic pressure (AoD) were all calculated using normal conventions. The effect of calcium on hemodynamics was clearly observable in most cases and the physiological effect could be approximated using a sigmoid shape. However, the heterogeneity of the mean blood pressures and their slope during recovery from pseudo-PEA would have obscured any analysis of the raw data. To understand the size of the pressor effect of calcium, blood pressure data were normalized such that the midpoint of observed effect occurred at a pressure of 0 mmHg and a time of 0 s. This normalization was performed by a second python script which used the zero-crossing point in the second derivative of the systolic aortic pressure, the second derivative of the coronary perfusion pressure, and the second derivative of the cerebral perfusion pressure to approximate the midpoint of the sigmoid response to a calcium bolus. Once the midpoint was found, pressures were normalized by subtracting the pressure value at the midpoint, and time was normalized by subtraction of the time when the midpoint occurred. Once the MAP, AoS, and AoD pressures were normalized, the mean values and standard deviations were calculated for the times −40 s < *t* < 40 s.

### Statistical methods

The normality of the mean aortic pressure, systolic aortic pressure, and diastolic aortic pressure signals was verified using the Shapiro-Wilk test. The hemodynamics were compared from the time period −40 < *t* < −36 (before) and the time period 36 < *t* < 40 (after), where *t* represents the time normalized to the effect of IV administration of calcium. Measurements were averaged across these two 4 s periods and compared via a paired *t* test (STATA v 15.1). The serum baseline, post-round 1, and post 2+ round ionic calcium concentrations were aggregated between cohorts 1 and 2. The normality of the aggregate initial iCa, post-round 1 iCa, and average remaining post 2+ rounds iCa was verified using the Shapiro-Wilk test. These measurements were compared via one-way ANOVA (Excel v. 16.38).

## Results

Tables 1 and 2 demonstrate the blood chemistry results for all pigs from each cohort. Blank entries indicate that the animal did not survive to that round, whereas “missing” indicates incomplete or missing data. One-way ANOVA analysis of the aggregate data for animals who did not receive bolus calcium (cohorts 1 and 2, Table 3) shows significant decreases in baseline, post-round 1, and subsequent rounds iCa (*p* = 0.03). In cohort 1, some animals did not require CPR for resuscitation, however, there was no significant difference in iCa between pigs who did or did not require CPR (*p* = 0.89). Animals in cohorts 2 and 3, all received CPR.

**Table 3:** Results of blood chemistries from cohorts 1 and 2 that did not receive calcium treatment

	Baseline (*N* = 24)		Round 1 (20 experiments)		Other rounds (43 experiments)	
	Mean	Std	Mean	Std	Mean	Std
pH	7.46	0.07	7.33	0.11	7.34	0.08
iCa [mmol/L]*	1.28	0.09	1.23	0.08	1.19	0.12

Improved pH between round 1 and other rounds are attributable to bicarbonate infusions during stabilization to prevent lethal acidemia

*One-way ANOVA between baseline, round 1, and other rounds demonstrated significant differences between the three groups (*p* = 0.03)

The blood chemistries of animals that received IV calcium are shown in Table 4 as a function of the experimental round. Because of the calcium treatments, measured iCa values were not different between the rounds. Base excess (BE), bicarbonate (HCO3), and total carbon dioxide (TCO2) were lower after PEA than at baseline but were not affected by subsequent pseudo-PEA injuries.

**Table 4:** Results of blood chemistries from the 9 animals that received iCa in cohort 3

	Baseline		Round 1 (*N* = 9 rounds)		Other Rounds (*N* = 15 rounds)	
	Mean	Std	Mean	Std	Mean	Std
pH	7.45	0.024	7.22	0.14	7.31	0.077
PCO2 [mmHg]	44.42	3.30	52.48	7.04	47.23	10.62
pO2 [mmHg]	238.67	57.67	329.78	154.50	365.4	119.59
BEecf [mmol/L]	7	1.73	−16	31.40	−2.67	2.23
HCO3 [mmol/L]	31.06	1.62	21.57	4.34	23.54	1.87
TCO2 [mmol/L]	32.22	1.64	23.33	4.21	24.87	1.92
sO2%	99.88	0.33	96.89	8.96	99.93	0.26
Na [mmol/L]	137.55	1.88	140.22	9.90	139.87	2.26
K [mmol/L]	4.2	0.23	3.79	0.43	4.12	0.62
iCa [mmol/L]	1.3867	0.05	1.22	0.14	1.33	0.15
Glu [mg/dL]	83.22	23.75	213.11	127.61	152	85.83
HCT [% PCV]	23.22	3.27	32.44	3.81	30.13	4.31
HB [g/dl]	7.911	1.13	11.03	1.31	10.25	1.47

*Improved base excess, pCO2, and pH between round 1, and other rounds are attributable to bicarbonate infusions during stabilization to prevent lethal acidemia

**Fig. 1:**
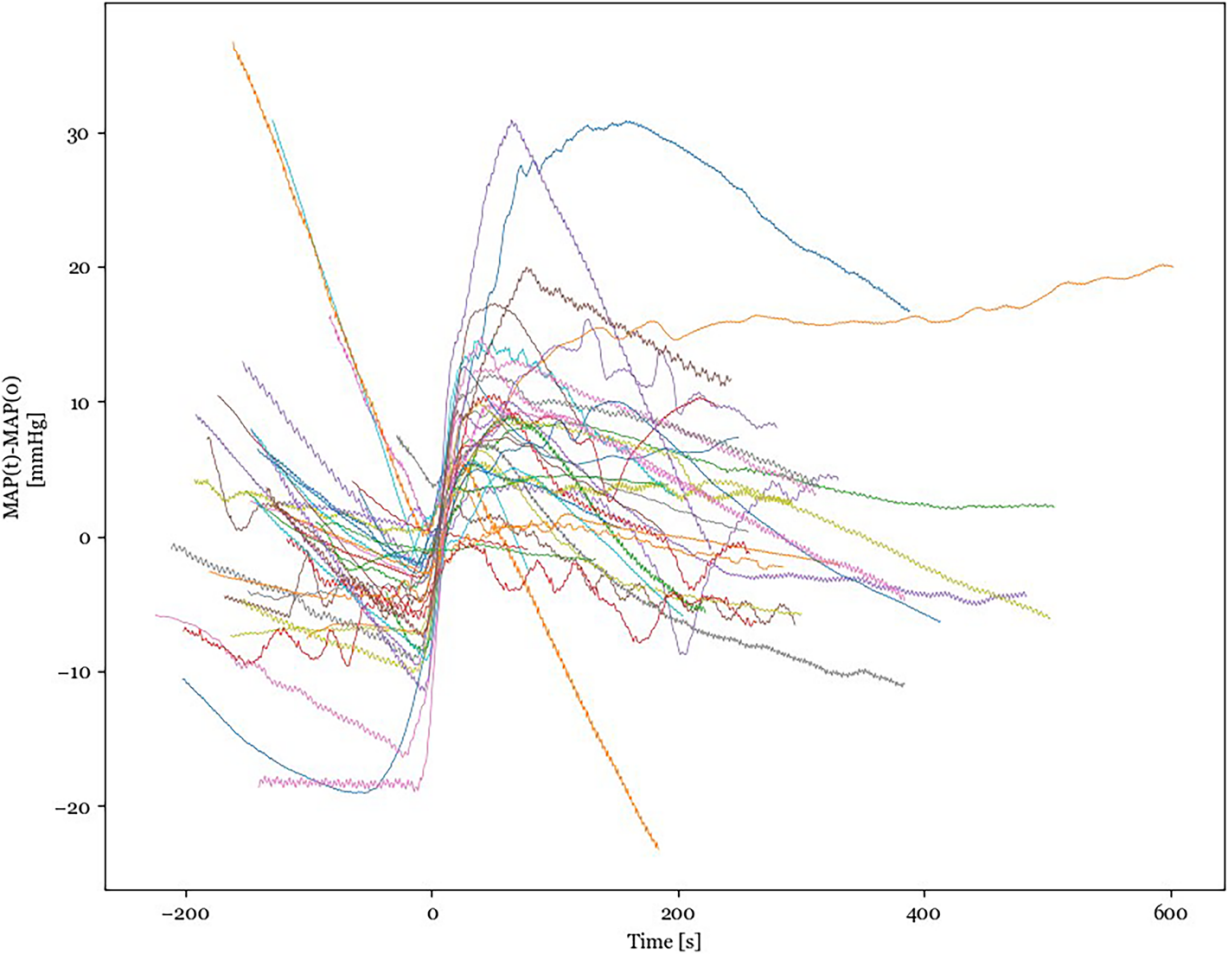
Normalized mean arterial pressure tracings are shown for all 37 boluses of calcium. Displayed pressures have been smoothed using a 15-sample windowed average to reduce the ventilation artifact. There are a variety of physiological responses to a bolus of calcium

Calcium doses were delivered in the range of 5-20 mg with the majority of the doses being 10 mg. In total, 37 unique boluses of calcium were annotated in the experimental record. The normalized aortic blood pressures from all 37 boluses are shown in Fig. 1. The data in this figure have been smoothed to reduce the ventilation artifact so that the individual responses are easier to discern. The different responses in Fig. 1 demonstrate the hyperdynamic nature of the hemodynamics after resuscitation from pseudo-PEA. The effect of the calcium bolus is observed before time 0 because time 0 was set to the midpoint of the pressor effect. Because the amount of data exported for each delivered calcium bolus depended on the time interval between the calcium bolus of interest and the injection of other medications such as epinephrine, bicarbonate, and other doses of calcium, the different lengths of the data segments also demonstrate the variability in the amount of hemodynamic support the animals needed after resuscitation.

**Fig. 2:**
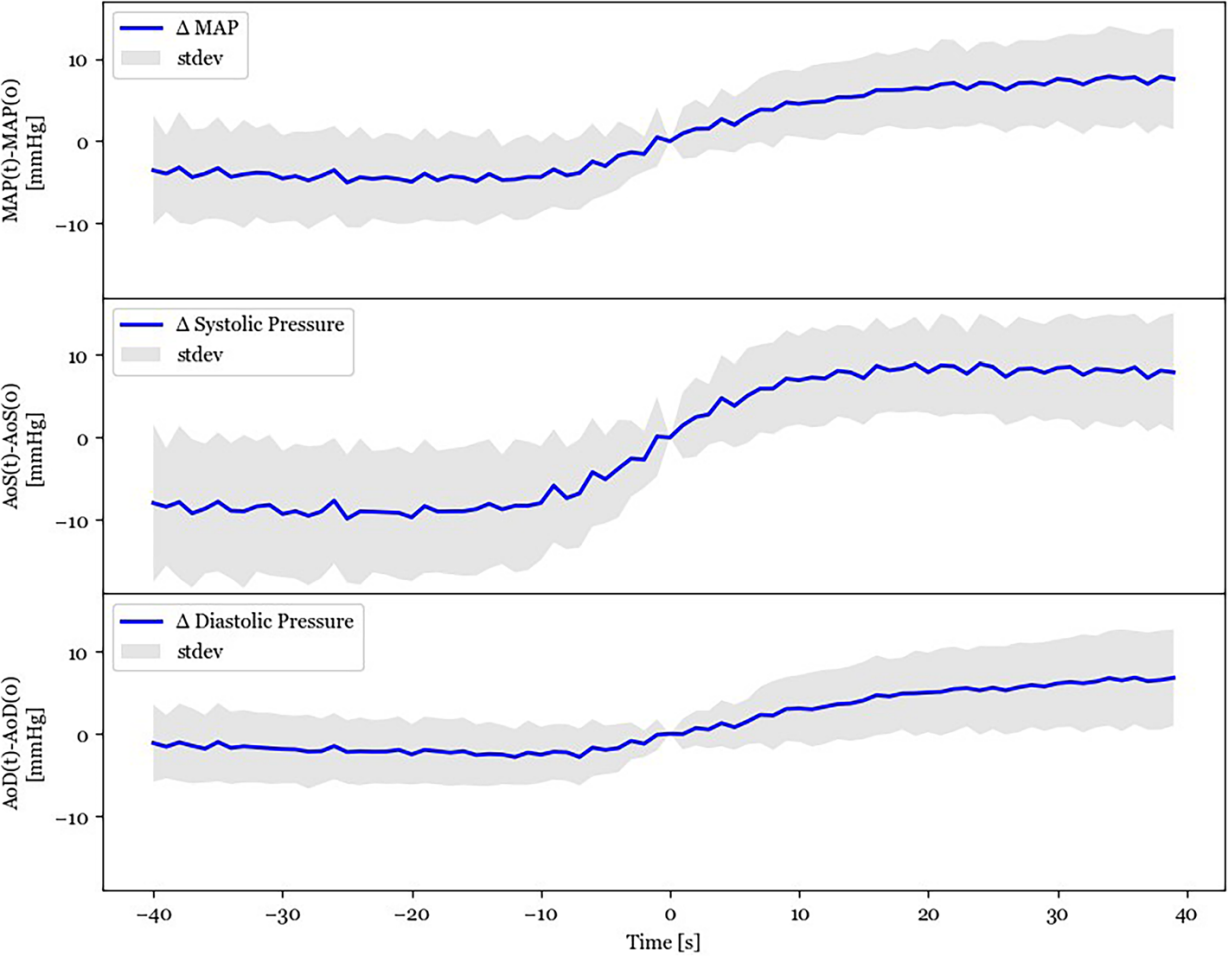
In each subplot, mean normalized arterial pressures are shown with a blue line, and the gray shaded area represents the standard deviation of the same pressures. The top subplot shows the effect of calcium on the mean aortic pressure. The middle subplot shows the effect of calcium of the systolic aortic pressure. The bottom subplot shows the effect of calcium on the diastolic aortic pressure. A calcium bolus results in a sigmoidal increase in arterial pressures. Time 0 is the midpoint of the pressor effect as determined by computer script. The pressures have been normalized by the subtraction of the pressure measured at time 0

To compare the hemodynamics before and after the calcium bolus, we chose timespan of [−40 s < *t* < 40 s ] for which data are present in all animals. Mean aortic pressures (MAP), systolic aortic pressure (AoS), and diastolic aortic pressure (AoD) were averaged as a function of time. The mean aortic pressure responses are represented by the blue line in Fig. 2. The shaded region above and below the blue line represents the standard deviation of those values. The mean pressures and the standard deviation of the pressures approaches zero as the time approaches zero due to the normalization procedure discussed in the methods. Comparing blood pressures from the timespan [−40 s < *t* < −36 s] with blood pressures from the timespan [36 s < *t* < 40 s], we find that the MAP increased by 12.7 ± 6.5 mmHg (*p* < 0.05), the AoS increased by 10.6 ± 8.5 mmHg (*p* < 0.05), and the AoD increased by 3.1 ± 4.5 mmHg (*p* < 0.05).

**Fig. 3:**
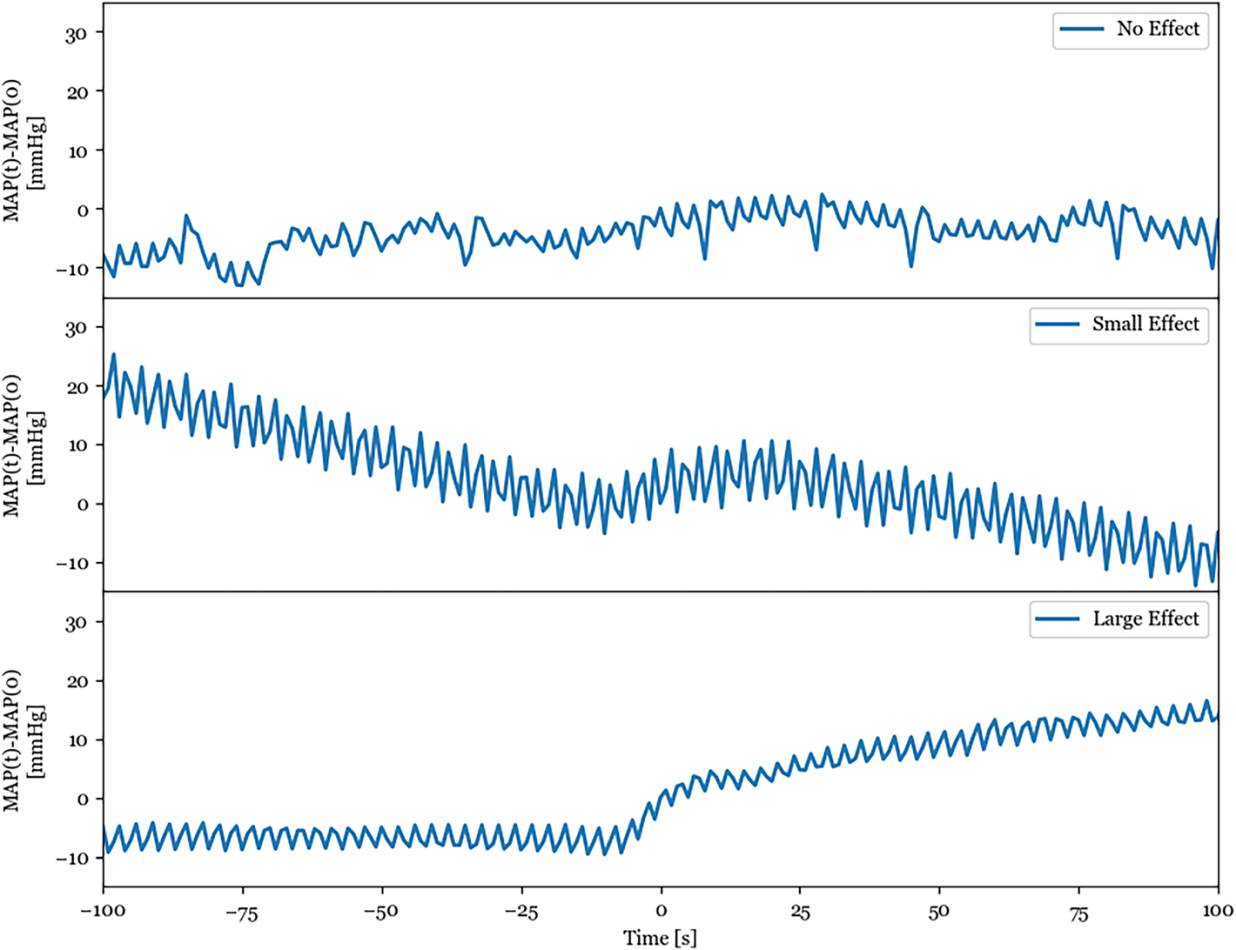
There are a variety of individual responses to a calcium bolus. The top subplot shows a calcium injection that has no observable effect on the mean arterial pressure. The middle subplot shows a calcium injection that has a modest effect on the mean arterial pressure, but the MAP resumes its decline after about 20 s of improvement. The bottom subplot shows a calcium injection that has a large effect on arterial pressure where the MAP improves due to the calcium bolus and then continues to improve and stabilize as a function of time. These tracings were chosen to represent the spectrum of observed responses

**Fig. 4:**
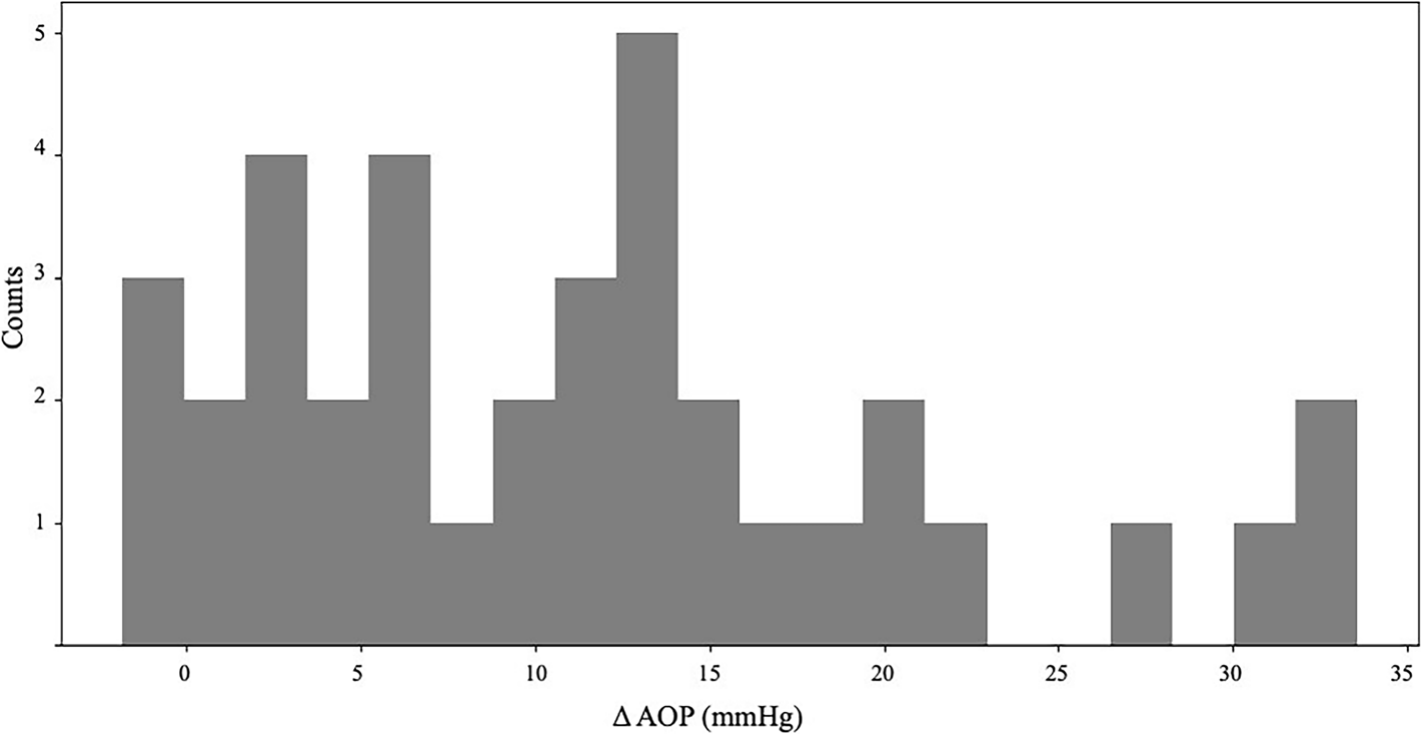
This histogram of the change in mean aortic pressure was calculated by subtracting the mean aortic pressure 36-40 s before the calcium bolus from the mean aortic pressure 36-40 s after the calcium bolus. While most of these interventions are associated with a positive change in mean aortic pressure, in four experiments, the mean aortic pressure decreased. In these experiments, the animal was decompensating rapidly, and the continuing drop in blood pressure was larger than the benefit of the calcium bolus

The physiological response to the calcium boluses manifested in a range of MAP responses, as shown in Fig. 3. In Fig. 4, the histogram shows the range of changes in the MAP for all 37 boluses. In four experiments, the MAP 40 s after the bolus was lower than the MAP 40 s before the bolus.

## Discussion

Our preliminary results indicate that similar to ventricular fibrillation, ionized hypocalcemia may be common after resuscitation from pseudo-PEA, and the associated refractory hypotension may be responsive to administration of IV calcium. Specifically, administration of calcium in this setting is often associated temporally with a relatively rapid pressor response. Of note, calcium administration was also often associated with a stabilization of blood pressure when it had been previously trending downward. In 16 instances, this stabilization was maintained until the end of that cycle in the experiment. In the remainder of cases, bolus calcium either resulted in only a temporary pressure increase followed by a return to progressive decline or no measurable effect. In the four instances, where MAP 40 s after the bolus was lower than the MAP 40 s before, the MAP was decaying rapidly and the ongoing rapid loss of blood pressure was larger than the benefit of the calcium bolus, resulting in a negative pressure change. In these cases, it might be inaccurate to assume that the treatment effect resulted in a decrease in blood pressure or that the effect was marginal.

The relationship between ionized calcium and critical cardiovascular states is not fully understood [4]. Prior reports describe hypocalcemia in other cardiac arrest states [11]. Of the metabiolic abnormalities in these other hypotensive states, it was noted that there was often a relationship between ionized calcium concentration and pH. Proposed explanations of this phenomena include intracellular calcium influx from ion compartment shifting, changes from impaired transmembrane pump activity, and extracellular protein complexing. Under ischemic conditions, it has been demonstrated that cells increase their uptake of calcium as a result of altered membrane transport; moreover, intracellular and mitochondrial calcium sequestration is an important initiator of apoptosis [12]. It is likely that a combination of these factors may contribute to an overall depletion in ionic calcium stores in pseudo-PEA. Ischemia and the resultant loss of normal cellular energy state seem likely to underlie these pathophysiologic processes.

Hypocalcemia likely also contributes to the hypotension that is common in the recovery period of pseudo-PEA. Both cardiac and vascular smooth muscles are dependent on calcium for function. In cardiac myocytes, calcium-dependent calcium release is a critical component initiating robust ventricular contraction; hypocalcemia is a known cause of negative inotropy and dysrhythmia. For vascular smooth muscle, calcium is needed to activate calmodulin to allow for fiber crosslinking. Effective calcium starvation impairs vasomotor tone and results in a decrease in peripheral resistance. It is thus not surprising that hypotension after pseudo-PEA is often refractory to catecholamine pressors. Functional calcium deficits in both the myocardium and vascular smooth muscle likely blunt the sympathetic stimulation. Diffuse hypoxic insult with associated metabolic derangement may underlie continued hypocalcemia even after ROSC is achieved.

Hypotension after resuscitation from profound global hypoxia and ischemia, as in our porcine model of pseudo-PEA, is a particularly challenging cardiovascular state for clinicians. Leaving it untreated may worsen outcome through secondary injury, as in a “two-hit” model. As noted before, it is often refractory to catecholamine pressers [1], which may themselves be associated with a worse outcome [12]. It was relatively common during the 1960s and 1970s to treat cardiac arrest with boluses of IV calcium without evidence of efficacy [13]. This has become much less common in the era of guideline-based advanced life support.

Pseudo-PEA is common clinically—possibly present in half of all arrests—and outcomes are often poor [14]. It has not been studied to the same extent as ventricular fibrillation. The pseudo-PEA cardiovascular state can be created by hypoxia, asphyxia, cardiotonic drug overdose, and post-defibrillation [15]. Unfortunately, it has been difficult to create stable models using these insults. Our model is relatively stable and reproducible. However, the hypoxic insult in juvenile pigs may be most representative clinically of pediatric partial asphyxiation [15], such as is seen in asthma, and not the PEA caused by coronary occlusion in adults. For this reason, the applicability of our model to adult patients suffering ischemic cardiac arrest may be limited.

Although collected retrospectively, our preliminary data indicates that ionized hypocalcemia may be present after resuscitation from pseudo-PEA and that bolus IV calcium is associated with a sustained pressor response. Intriguingly, this pressor effect often stabilized progressively declining blood pressure. These finding should be investigated prospectively. If confirmed, they may justify a clinical trial of intravenous calcium in the setting of pseudo-PEA or hypotension after resuscitation from pseudo-PEA.

However, it should be emphasized that hypotension is a physiologic biomarker and not a clinical outcome per se. It is possible that administration of intravenous calcium temporarily improves blood pressure but then is associated with an overall worse clinical outcome because it enhances vital organ injury by priming of apoptosis [16]. It is important that clinicians not respond to this preliminary post hoc data set by empiric administration of IV calcium to patients suffering pseudo-PEA. A clinical trial with a primary endpoint of improved neurologically intact long-term survival would need to be completed preliminary to a change in clinical practice.

Limitations—our report has a number of limitations: (1) this is an animal model of hypoxic pseudo-PEA, (2) the porcine model is juvenile and without senescent comorbidities, (3) intravenous calcium levels and the blood pressure response to calcium administration were not the primary processes under study, these are retrospectively observed results, (4) the before/after comparison used does not necessarily capture the effect of the calcium bolus, as in the four cases where MAP decreased due to rapid decompensation, (5) improved MAP is not a clinical outcome.

## Conclusion

Our preliminary results indicate that the hypotension after hypoxia-induced pseudo-PEA is frequently associated with decreased serum ionized calcium levels and a hypotension that is responsive to administration of IV calcium. These results should be confirmed prospectively before consideration is given to clinical trials.

## Data Availability

The authors declare that all data supporting the findings of this study are available within the article.

## Abbreviations

ACLS: Advanced cardiac life support
AoD: Diastolic aortic pressure
AoS: Systolic aortic pressure
BE: Base excess
CPR: Cardiopulmonary resuscitation
EMD: Electro mechanical dissociation
ETCO2: End-tidal carbon dioxide
FiO2: Inspired fraction of oxygen
HCO3: Bicarbonate
iCA: Ionic calcium
IV: Intra-venous
pseudo-PEA: Pseudo pulseless electrical activity
MAP: Mean arterial pressure
N2: Nitrogen
O2: Oxygen
pCO2: Partial pressure of carbon dioxide
PEA: Pulseless electrical activity
P-EMD: Pseudo-electromechanical dissociation
pO2: Partial pressure of oxygen
ROSC: Return of spontaneous circulation
TCO2: Total carbon dioxide
